# 

**Published:** 1871-12

**Authors:** 


VOL. I. DECEMBER 1871. NO. 12.

### THE GEORGIA MEDICAL COMPANION,

DEVOTED TO THE

### SCIENCE OF MEDICINE AND SURGERY.

EDITORS:

T. S. POWELL, M. D., and W. T. GOLDSMITH, M. D.

ASSOCIATE EDITORS:

GEORGIA.

Robert Battey, M D,

R C Word, M D,

W L Kirkpatrick, M D,

James A Long, M D,

W L M Harris, M D,

H L Battle, M D,

W C Musgrove, M D,

L B Burchelle, M D,

John C Drake, M D,

E F Knott, M D,

Thomas Burdell, M D,

E H W Hunter, M D,

V G Hitt, M D,

J E Pope, M D,

C H Bass, M D,

J A Hunnicutt, M D,

A H Brantly, M D,

J J Knott M D,

ALABAMA.

J C Knox, M D,

Benj H Riggs, M D,

Geo F Taylor, M D,

J B Barnett, M D,

S M Hogan, M D.

MISSISSIPPI.

C B Gallaway, M D,

Edward Lea, M D,

S V D Hill, M D,

W B Harvey, M D,

J W Shattuck, M D,

E T Henry, M D,

T P Lockwood, M D,

Robert Kells, M D.

A B Pierce, M D, N. C.

VIRGINIA.

Charles S Lewis, M D,

John H Claiborn, M D,

C R Harris, M D,

F Horner, Jr, M D,

A S Payne, M D,

R J Preston, M D,

J S Whorton, M D.

Wm O Owen, M D,

Sam'l P Ripley, M D,

Benj Blackburn, M D,

E A Drewry, M D,

Rob. B Tunstall, M D,

A J Terrell, M D,

Wm F Barr, M D,

J M Lazzell, M.D.

E. F McSwain, M D, S C

CORRESPONDING EDITORS:

J M Toner, M D, Washington City, D C; John H Weir, M D, Ill; Thomas J Gallaher, M D, Pa; Eli Van DeWarker, M D, N Y; Robert Robson, Ind; C C F Gay, M D, N Y, (Surgeon of Buffalo General Hospital); W S Taylor, M D, Ky; A Patton, M D, Ind; J Orne Green, Boston, Mass; B W Stone, M D, Ky, (Assistant Physician Western Lunatic Asylum); James Tyson, M D, Philadelphia, Pa; M H Henry, M D, N Y, (Surgeon to New York Dispensary, Department of Venerial and Skin Diseases); Wm R Whitehead, M D, N Y; Thomas H Kearney, M D, Cincinnati, O; Benjamin Howard, M D, New York, Chas Kearns, M D, Ky, S C Busery, M D, Washington, D C.

*"QUICQUID PRÆCIPIES ESTO BREVIS."*

ATLANTA, GA.
FRANKLIN STEAM PRINTING HOUSE.
J. J. TOON, PROPRIETOR.

TERMS, - - - - $2 A YEAR, IN ADVANCE.

